# Epidemiology of sudden death in organized school sports in Japan

**DOI:** 10.1186/s40621-021-00326-w

**Published:** 2021-07-12

**Authors:** Yuri Hosokawa, Yuki Murata, Rebecca L. Stearns, Miwako Suzuki-Yamanaka, Kristen L. Kucera, Douglas J. Casa

**Affiliations:** 1 Faculty of Sport Sciences, Waseda University, 2-579-15 Mikajima, Tokorozawa, Saitama Japan; 2 Graduate School of Education and Human Development, Nagoya University, Nagoya, Aichi Japan; 3 Korey Stringer Institute, Department of Kinesiology, University of Connecticut, Storrs, CT USA; 4 Graduate School of Sport Sciences, Waseda University, Tokorozawa, Saitama Japan; 5 Department of Exercise and Sport Science, University of North Carolina Chapel Hill, Chapel Hill, NC USA

**Keywords:** Catastrophic injury, Sudden cardiac arrest, Head trauma, Exertional heat stroke, School athletics

## Abstract

**Background:**

Nearly half of the sudden deaths documented in Japanese middle and high school occurred during school organized sport activities. However, no study to date has calculated the incidence rates of these deaths by sport. Therefore, this study aimed to describe the epidemiology of sudden death in organized school sports in Japan.

**Methods:**

Data submitted to Japan Sport Council (JSC) Injury and Accident Mutual Aid Benefit System between 2005 and 2016 were retrieved from JSC website for analysis (*n* = 1137). Case information on fatal incidents that occurred during organized school sports in middle and high school students were extracted for analysis (*n* = 198). Descriptive statistics about activity type, sex, sport, cause of death, and presence of on-site trained medical personnel were calculated using frequencies and proportions. Sudden death incidence rates were expressed per 100,000 athlete-years with 95% confidence intervals (CI).

**Results:**

The overall incidence rate of sports-related death was 0.38 deaths per 100,000 athlete-years (95%CI = 0.30, 0.45). Only three cases (2%) reported having trained medical personnel on-site at the time of death. Most deaths were in male student athletes (*n* = 149/162, 92%), with 7.5 times greater fatality rate in male compared to female student athletes (incidence rate ratio, 7.5; 95%CI = 4.43, 13.22). Baseball (*n* = 25/162, 15.4%), judo (*n* = 24/162, 14.8%), soccer/futsal (*n* = 20/162, 12.3%), and basketball (*n* = 18/162, 11.1%) accounted for 53.7% of deaths. Accounting for the number of participants in the respective sport, the three highest average incident rates of death were reported in rugby (4.59 deaths per 100,000 athlete-years, 95%CI = 2.43, 6.75), judo (3.76 deaths per 100,000 athlete-years, 95%CI = 1.58, 5.93), and baseball (0.59 deaths per 100,000 athlete-years, 95%CI = 0.38, 0.79). The top three causes of death were sudden cardiac arrest (*n* = 68/162, 42.0%), head trauma (*n* = 32/162, 19.8%), and heat related injury (*n* = 25/162, 15.4%).

**Conclusions:**

In conclusion, the highest rates of sports-related death among Japanese student athletes were observed in the following: rugby, male athletes, and during practices. The leading cause of death was sudden cardiac arrest.

## Background

School organized sport participation provides a valuable opportunity for students to stay physically active, start a new sport, and fosters psychosocial growth in the form of teamwork, leadership, and accountability. Japan Sports Agency and Japanese school systems have regarded school organized sport as an extension of formal education that is supervised and coached by faculty members of the school (Japan Sport Agency 2018; Nakazawa 2014). Participation in school organized sport is often viewed as an introduction to an active lifestyle to extend a healthy life span (Hallal et al. 2006; Monma et al. 2019); however, it could also predispose student athletes to sustain injuries that may range from minor to fatal (Kamiya et al. 2015; Kuroyanagi et al. 2001; Miyake et al. 2016; Takagishi et al. 2019; Takahashi et al. 2019).

Death of a student athlete during school organized sport not only impacts the victim’s family and team, but the community at large. According to Japan Sport Council (JSC) Injury and Accident Mutual Aid Benefit System, middle and high schools documented an average of 360 claims for catastrophic and fatal incidents each year from school sanctioned activities from 2015 to 2018, 46% of which were related to sport participation (outside of physical education) (Japan Sport Council n.d.-a). School nurse may be on call afterschool when school organized sports take place; however, the level of access varies greatly by school. In addition, current school safety guidelines set forth by the Ministry of Education, Culture, Sports, Science and Technology focus on community safety, traffic safety, and natural disaster safety but not sports-specific safety considerations. Therefore, school administration, staff, and faculty are not equipped with knowledge and skills regarding prevention and management of sport-specific emergencies. Consequently, when fatal sports incidents do occur, they are managed in a reactive fashion, where cases are examined retrospectively to identify the mechanisms and causes of death through interviews, but often fall short on implementing proactive risk mitigation strategies. At the time of the current study, no nationwide policy has specifically aimed to improve health and safety of student athletes in the context of school organized sport participation.

Each year, JSC Injury and Accident Mutual Aid Benefit System receives insurance claims data from schools for catastrophic and fatal incidents that occurred during school sanctioned events (Japan Sport Council n.d.-a). The mutual aid benefit system covers medical expenses and compensation for injuries or death, if the accident that results in injury, illness, disability, or death occurred under the supervision of schools. The liability for the premium is borne by the national government, school, and parents and guardians, and eligibility for benefits applies regardless of the school’s liability. After the student-athlete is discharged from the hospital, parents and guardians will submit necessary medical record information to the school, who will process the mutual aid benefit system application to JSC. The benefit will be paid out to the parents and guardians of the insuree if the claim application is submitted to JSC within 2 years of the incident. Ninety-five percent of the students of both public and private institutions nationwide (e.g., compulsory education schools, high schools, kindergarten, nursery school) subscribe to this mutual aid benefit system with 99.9 and 97.8% of middle and high school students, respectively, covered under this system (Japan Sport Council n.d.-b). No study to date has examined the incidence of sport-related death using the current dataset.

## Methods

A retrospective epidemiology study design was used to describe the epidemiology of sudden death in organized middle school and high school sports in Japan.

### Data sources

Insurance claims submitted to JSC Injury and Accident Mutual Aid Benefit System between 2005 and 2016 were retrieved from JSC website for analysis (*n* = 1137). The database is publicly accessible on the website (Japan Sport Council n.d.-a). Case information on fatal incidents occurring during organized school sports in middle and high school students were extracted by YH for further analysis (*n* = 198). In Japan, middle school covers grades 7th through 9th (12 to 15 years old) and high school covers grades 10th through 12th (15 to 18 years old). Data categorization used in the JSC database was used in this study for: activity type (practice, scrimmage, game, training camp, other), sport (badminton, baseball, basketball, boxing, cycling, fencing, gymnastics, handball, hiking, ice hockey, judo, kendo, kyudo, rugby ski, soccer, softball, swim table tennis, track and field [long distance, pole vault, short distance, throwing], volleyball, wrestling, other) and cause of death (cervical spine trauma, drowning, head trauma, heat related injury, internal organ trauma, lightning, sudden cardiac arrest, suffocation, and unknown cause). Cause of death was registered in the database by JSC staff according to the medical diagnosis provided by the physician in the medical records submitted by the insuree.

Population data for middle and high school student athletes were retrieved from public online databases of national governing bodies that manage the membership data of these athletes annually (All Japan High School Athletic Federation, 2017; Nippon Junior High School Physical Culture Association n.d.-a; Nippon Junior High School Physical Culture Association n.d.-b). Due to missing data in some years from the publicly available data source, data from 2016 was used as the estimate of participant numbers in all years. Since high school baseball is governed by Japan High School Baseball Federation, membership data was retrieved separately from its official website (Japan High School Baseball Federation n.d.).

### Data analysis

Descriptive statistics related to information about activity type, sex, sport, cause of death, and presence of on-site trained medical personnel were calculated using frequencies and proportions. Presence of on-site trained medical personnel was determined by YH from reviewing case details. Sudden death incidence rates were expressed per 100,000 athlete-years (AY) with 95% confidence intervals (CI). Incidence rates overall and by gender were calculated using the total number of middle and high school student athletes in the rate denominator. Incidence rate ratio and 95% CI between male and female student athletes was also calculated. Sport-specific incidence rates were calculated for sports with five or more reported deaths, and utilized the number of participants in the sport in the rate denominator. Fisher’s exact test (FET) with Monte Carlo simulation was used to examine the pattern in the proportion of reported causes of death (sudden cardiac arrest, head trauma, heat related injury, and all other causes of death) over the 12-year period. A significance level of *p* < 0.05 was set a priori.

## Results

Fatalities were reported during practice (*n* = 120/198, 60.6%), scrimmage (*n* = 16/198, 8.1%), game (*n* = 10/198, 5.1%), training camp (*n* = 16/198, 8.1%), and other (*n* = 36/198, 18.2%). For the remainder of the analysis, fatalities that occurred during *other* activities were removed from the analysis since they either (1) did not have further details to suggest that they occurred during practice, scrimmage, game, or training camp, or (2) the report suggested that they occurred during the downtime of sport participation (e.g., found dead in bed the morning after the practice, motor vehicle accident on the way to athletic venue).

The overall incidence rate of sports-related death was 0.38 deaths per 100,000 AY (highest, 0.56 deaths per 100,000 AY; lowest, 0.19 deaths per 100,000 AY). Most fatalities were in male student athletes (*n* = 149/162, 92%), with a 7.5 times greater fatality rate (95%CI = 4.43, 13.22) in male compared to female student athletes (male, 0.60 deaths per 100,000 AY, 95%CI = 0.46, 0.73; female, 0.08 deaths per 100,000 AY, 95%CI = 0.03, 0.13). Only three deaths (2%) reported having trained medical personnel on-site at the time of incident.

### Analysis by sport

**Fig. 1:**
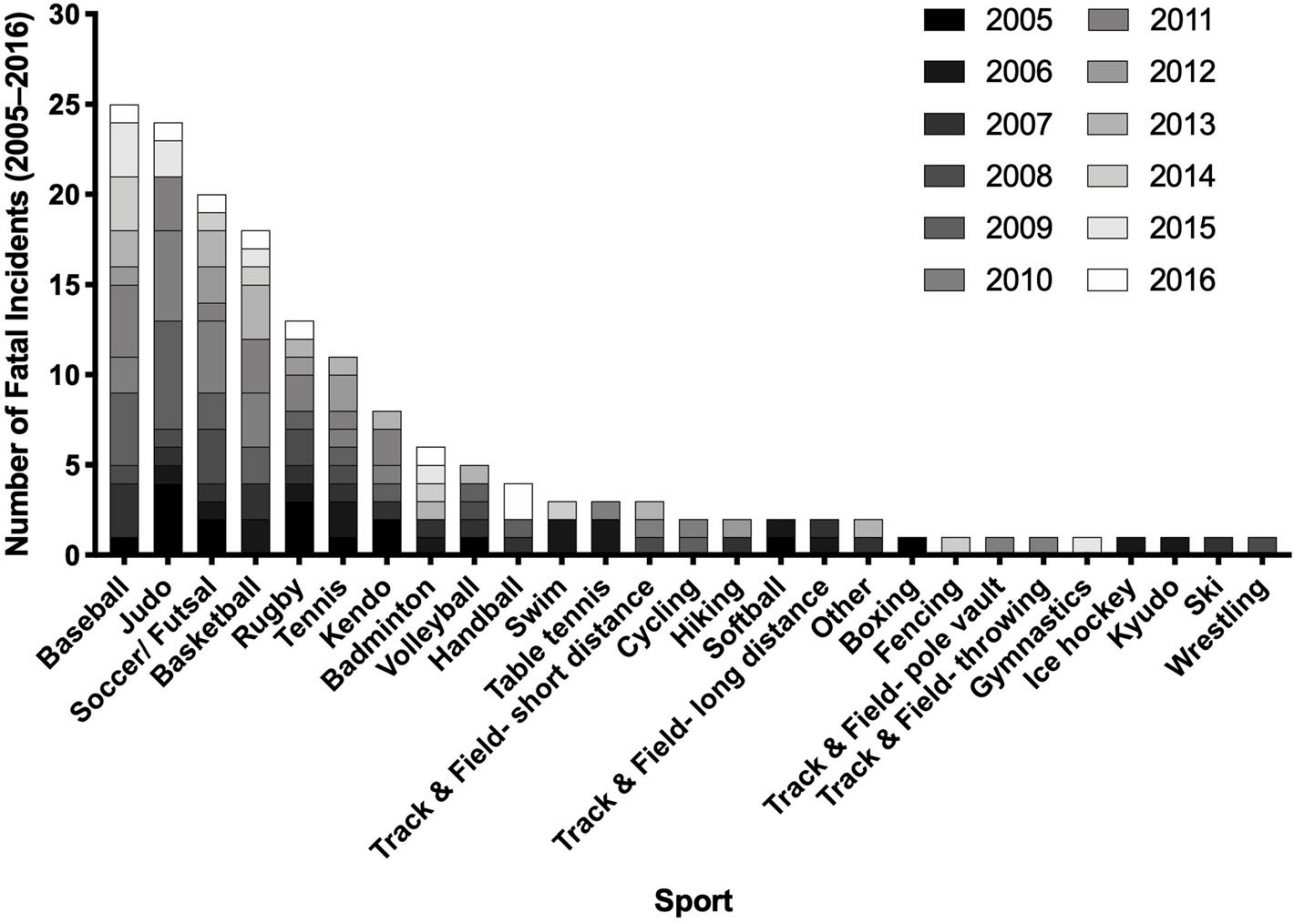
Number of fatal incidents reported during organized school sports in Japanese middle and high school between 2005 and 2016 by sport

The number of fatalities by sport is summarized in Fig. 1. Baseball (*n* = 25/162, 15.4%), judo (*n* = 24/162, 14.8%), soccer/futsal (*n* = 20/162, 12.3%), and basketball (*n* = 18/162, 11.1%) accounted for 53.7% of fatalities. Incidence rates per 100,000 AY were calculated for sports with five or more reported deaths (e.g., baseball, judo, soccer/futsal, basketball, and rugby, tennis, kendo, badminton, and volleyball). Accounting for the number of participants in the respective sports, the three highest incident rates of death were reported in rugby (4.59 deaths per 100,000 AY, 95%CI = 2.43, 6.75), judo (3.76 deaths per 100,000AY, 95%CI = 1.58, 5.93), and baseball (0.59 deaths per 100,000AY, 95%CI = 0.38, 0.79) (Table 1).

**Table 1:** Overall incident rates of death during organized school sports in Japanese middle and high school by sport between 2005 and 2016

Sport	Number of deaths	Number of athletes	Incident rate per 100,000 athlete-years	95%CI	
Rugby	13	283,224	4.59	2.43	6.75
Judo	24	638,340	3.76	1.58	5.93
Baseball	25	4,264,644	0.59	0.38	0.79
Kendo	8	1,600,332	0.50	0.17	0.83
Soccer/ Futsal	20	4,972,080	0.40	0.26	0.55
Basketball	18	5,620,116	0.32	0.18	0.46
Badminton	6	2,975,640	0.20	0.08	0.32
Tennis	11	6,604,788	0.17	0.10	0.24
Volleyball	5	3,852,084	0.13	0.04	0.22

Population data for middle school and high school student athletes were retrieved from the Nippon Junior High School Physical Culture Association and All Japan High School Athletic Federation membership data (middle school, http://njpa.sakura.ne.jp/pdf/kamei/h28kameiseito_m.pdf and http://njpa.sakura.ne.jp/pdf/kamei/h28kameiseito_f.pdf; high school, https://www.zen-koutairen.com/pdf/reg-28nen.pdf). Due to missing data in some years, data from 2016 was used as the estimate of participant numbers in all years

### Cause of death

**Fig. 2:**
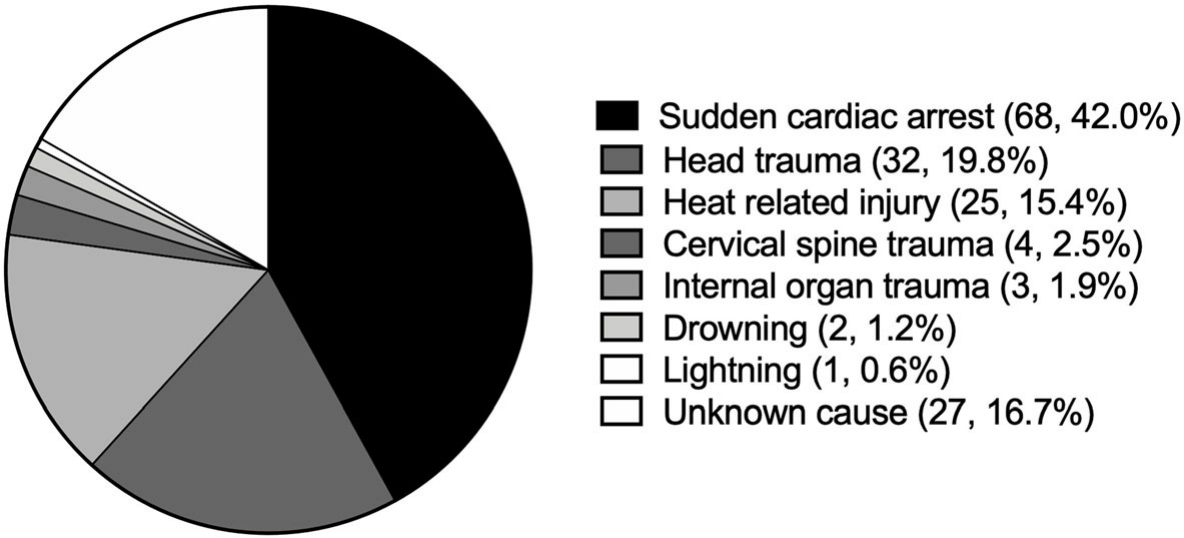
Cause of death reported during organized school sports in Japanese middle and high school between 2005 and 2016

**Fig. 3:**
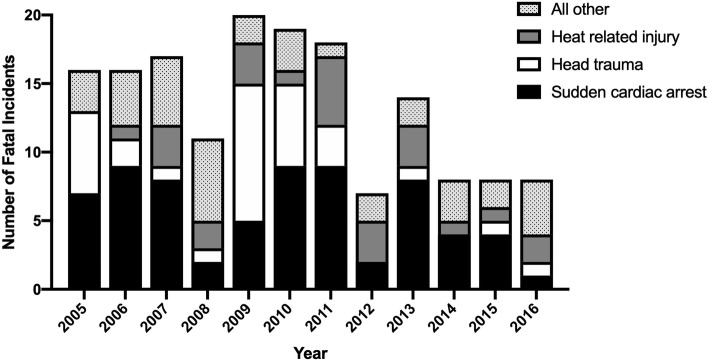
Number of fatal incidents reported during organized school sports in Japanese middle and high school by year and cause between 2005 and 2016

The cause of death during organized school sports is summarized in Fig. 2. Sudden cardiac arrest (*n* = 68/162, 42.0%), head trauma (*n* = 32/162, 19.8%), and heat related injury (*n* = 25/162, 15.4%) were the top three known causes of death. The proportion of sudden cardiac arrest, head trauma, heat related injury, and all other causes of death varied by year (FET [33] = 46.48, *p* = 0.02) (Fig. 3).

## Discussion

Examination of deaths that occurred during organized school sports documented in JSC database between 2005 and 2016 revealed that the overall incidence rate of sport-related death was 0.38 deaths per 100,000 AY, with 7.5 times greater rate in male compared to female student athletes. To our knowledge, this is the first study reporting the incident rate of death per 100,000 athlete-years during organized school sports in Japan. A previous study by Kuroyanagi et al. examined sudden deaths documented in elementary, middle, and high school students of the twenty-three special wards of Tokyo between 1976 and 1995 (Kuroyanagi et al. 2001). In their study, a total of 255 deaths were reported, of which 43 deaths happened during participation in school organized sports on campus. However, the incident rate per student-years was not calculated, and the sex difference was reported using a ratio (i.e., male to female ratio of reported death was 2 to 1), which does not provide a valid sex comparison since the total number of male participants was greater than that of female.

The three leading causes of death during organized school sports in Japan were comparable with those reported in the United States: sudden cardiac arrest, head trauma, and heat related injury (Boden et al. 2013; Casa et al. 2012b). This is an expected finding since sport-related deaths tend to occur during extreme exertion or from direct trauma (Boden et al. 2013). However, it should be noted that an ethnicity-based difference was observed in the absence of exertional sickling related death in our Japanese cohort; a risk factor commonly seen in people of African descent that is often associated with death when a training that is too novel, too much, too soon, or too intense is implemented (Casa et al. 2012a).

When accounting for the number of participants in the respective sport, rugby (4.59/100,000 AY) and judo (3.76/100,000 AY) had notably higher incidence rates of death compared to other sports. Rugby and judo were also the top two sports in reported numbers of head trauma deaths, and were the only two sports where the reported number of deaths due to head trauma exceeded sudden cardiac arrest (rugby: head trauma *n* = 6, sudden cardiac arrest *n* = 2; judo: head trauma *n* = 17, sudden cardiac arrest *n* = 1). This finding aligns with the high intensity and collision-involving nature of these sports. Recent American football injury research in the United States, from both media and direct reporting during a 12-year period, indicate that the incidence of sports-related deaths was 0.79 per 100,000 AY and 3.44 per 100,000 AY for high school and collegiate athletes, respectively (Kucera et al. 2020). Although these data cannot be used as a direct comparison against our study results due to differences in data collection methods, the findings in Japan were high because they exceed the rate observed in collegiate American football, which has the highest incidence rate of sports-related fatalities in collegiate sports in the United States.

There are two factors that may have affected a significantly high fatality rate reported in Japan. First, school organized sports in Japan are played throughout the academic year (April through March), 5 days a week. Furthermore, training camps, games, and tournaments are often scheduled during the holiday breaks, and students take part in the team activity for the duration of their time at the school (i.e., 3 years). Therefore, the total exposure to organized school activity is much greater in a given year compared to student athletes in the United States, where students participate in sports by seasons. Second, most school organized sports in Japan are solely supervised and coached by faculty members of the school (Nakazawa 2014). Before the introduction of external hiring policies (i.e., hiring non-faculty members) for school extracurricular activities in 2017, faculty members were assigned to sports that they may or may not be familiar with. The current analysis presented in this paper represents the pre-policy period, where such options were unavailable. According to the report from the Japan Sports Association, 45.9% of middle school and 40.9% of high school faculty members who were assigned to supervise school organized sport teams were not physical education teachers nor had the experience of playing the assigned sport (Japan Sports Association 2014). This is of concern as individuals with minimum, if any, knowledge of the sport and risks associated with the activity are responsible for the health and safety of the student athletes. While coaches are members of their respective sports governing bodies, the lack of regulatory systems for coaching qualifications at middle and high school sports makes it difficult to implement standardized coaching education as a requirement. According to the national survey conducted in 2015, 99% of middle and high schools in Japan have at least one automated external defibrillator on campus (The Ministry of Education, Culture, Sports, Science and Technology-Japan, 2015); however, the same survey reported that only 68.4 and 58.8% of middle and high schools offer first aid and cardiopulmonary resuscitation training to all faculty members. This discrepancy further highlights the lack of human resource and training to ensure the health and safety of student athletes. Lastly, only three reported cases in the current study had trained medical personnel on-site at the time of incident. This is in contrast with school organized sports in the United States, where 86% of athletes have access to certified athletic trainer for medical services (Pike et al. 2017). When a certified athletic trainer was present to assist in the resuscitation at the site of sudden cardiac arrest, 83% (24/29) of athletes survived; whereas the percentage was lower (47%; 9/19) when a certified athletic trainer was not present (Drezner et al. 2019). While we cannot imply causation from this finding, future improvement in access to trained medical personnel on campus may influence the number of sudden cardiac arrest sruvivals.

Sociocultural factors of Japanese school organized sports should also be considered in when interpreting results from the current study. A previous study (Mochizuki and Tomozoe 2016) suggests that origins of catastrophic injuries in Japanese school organized sports are due to lack of knowledge among coaches and use of physical hardship as a pedagogy for development. The zero tolerance culture for weakness has resulted in delay in appropriate activity modification and treatment when student athletes were suffering from potentially lethal conditions Future research should investigate organizational risk factors associated with catastrophic incidents during school organized sports to elucidate future strategies for prevention of sports-related fatalities.

Our dataset observed a drop in the number of deaths in 2008 and 2012 (Fig. 3). While we cannot make an inference, 2012 corresponds with the year when Judo became a required subject in physical education for boys and girls in middle school nationwide. This change has brought national attention to safety issues in judo, which heightened awareness regarding acquisition of proper skills and designing practice sessions that match the skill level of novice students. Detailed review of our dataset revealed that Judo had one fatal head trauma and one fatal cervical spine trauma from 2012 to 2016, which was substantially fewer than sixteen deaths reported between 2005 and 2011. This change in judo alone may have influenced the drop in the number of deaths from 2012 to 2016.

This study is not without limitations. First, authors retrieved the claims data from the publicly accessible database of JSC Injury and Accident Mutual Aid Benefit System, (Japan Sport Council n.d.-c) which is not designed for epidemiology research. For example, categories used to label causes of death in this system were created by JSC and do not follow code systems often seen in medical chart and injury surveillance systems. Additionally, the decision to submit claim information is made by school personnel, who may or may not have the ability to correctly identify deaths that occurred due to school organized sport participation. However, regardless of the shortcomings of the JSC database, it is the only national database that currently collects comprehensive claims reports on catastrophic and fatal injuries that resulted in insurance benefit payouts. Another limitation is our estimate of population data for rate denominators, which was derived from the membership data from 2016. This estimation does not account for changes in participation over time which could impact the rates. Furthermore, we did not have data on school characteristics to examine the confounding variables that may influence the chance of survival (e.g., presence of emergency action plans, accessibility to automated external defibrillator, number of years coaching the sport, completion of safety training). Lastly, future studies should examine factors that may have influenced the significantly low incidence rate among female student athletes. Previous studies have consistently reported a lower prevalence of sudden death among female athletes in sports; however, the precise mechanism is not well-understood (Colombo et al. 2019; Endres et al. 2019; Finocchiaro et al. 2016). Therefore, more studies are needed to improve the understanding of the causes of death among female athletes and to elucidate if different risk factors exist when compared to the male counterpart.

## Conclusions

In conclusion, the incidence rate of sports-related death among Japanese student athletes was highest in male rugby players, and fatalities most often occurred during practice and were due to sudden cardiac arrest. The top three causes of death were sudden cardiac arrest, head trauma, and heat related injury, accounting for 77.2% of all reported cases. Therefore, future efforts on prevention and emergency preparedness should prioritize these conditions. Additionally, few incidents had trained medical personnel on-site at the time of the catastrophic event during school organized athletics in Japan. These findings call for national and organizational actions to reconsider the current structure of school organized sports and to improve access to medical personnel during school organized sports.

## Data Availability

The dataset analyzed in the current study is freely available on Japan Sport Council Injury and Accident Mutual Aid Benefit System database at https://www.jpnsport.go.jp/anzen/anzen_school/anzen_school/tabid/822/Default.aspx.
